# Seasonal effects of influenza on mortality in a subtropical city

**DOI:** 10.1186/1471-2334-9-133

**Published:** 2009-08-22

**Authors:** Lin Yang, Chit Ming Wong, King Pan Chan, Patsy Yuen Kwan Chau, Chun Quan Ou, Kwok Hung Chan, JS Malik Peiris

**Affiliations:** 1 Department of Community Medicine and School of Public Health, The University of Hong Kong, 21 Sassoon Road, Hong Kong; 2 School of Public Health and Tropical Medicine, Southern Medical University, 1838 North Guangzhou Avenue, Guangzhou, PR China; 3 Department of Microbiology, The University of Hong Kong, 102 Pokfulam Road, Hong Kong; 4 HKU-Pasteur Research Centre, 8 Sassoon Road, Hong Kong

## Abstract

**Background:**

Influenza has been associated with a heavy burden of mortality. In tropical or subtropical regions where influenza viruses circulate in the community most of the year, it is possible that there are seasonal variations in the effects of influenza on mortality, because of periodic changes in environment and host factors as well as the frequent emergence of new antigenically drifted virus strains. In this paper we explored this seasonal effect of influenza.

**Methods:**

A time-varying coefficient Poisson regression model was fitted to the weekly numbers of mortality of Hong Kong from 1996 to 2002. Excess risks associated with influenza were calculated to assess the seasonal effects of influenza.

**Results:**

We demonstrated that the effects of influenza were higher in winter and late spring/early summer than other seasons. The two-peak pattern of seasonal effects of influenza was found for cardio-respiratory disease and sub-categories pneumonia and influenza, chronic obstructive pulmonary disease, cerebrovascular diseases and ischemic heart disease as well as for all-cause deaths.

**Conclusion:**

The results provide insight into the possibility that seasonal factors may have impact on virulence of influenza besides their effects on virus transmission. The results warrant further studies into the mechanisms behind the seasonal effect of influenza.

## Background

The impact of influenza on mortality has been well established in the temperate regions, but only recently has such impact been documented in tropical and subtropical regions [1,2]. Unlike in the cool temperate regions where influenza activity shows a distinctive seasonality with a well-defined influenza epidemic occurring almost every winter [3], in the warm tropics and subtropics seasonal patterns of virus activity are more diffuse, sometimes showing two influenza epidemics annually but with influenza viruses being isolated throughout the year [4].

Although influenza can be active in most time of the year, the upsurge of influenza viruses is not always followed by increased mortality [1]. We hypothesize that there are temporal variations in the effects of influenza on mortality. Such temporal variations are likely to exhibit a seasonal pattern because of seasonal variations in virus virulence and host susceptibility. Virulence of virus strains could be regulated through interaction between herd immunity, introduction of naïve individuals, and ability of virus to shed and to generate new strains [5]. The frequent genetic drift introduces novel virus strains to the population, which could pose a great threat at the beginning of circulation due to a lack of herd immunity [6]. Although impossible to predict when genetic drift occurs, such drift could result in temporal changes in virulence of circulating strains. Moreover, the environmental factors that favor transmission of influenza, such as low temperature and low humidity, appear in some seasons. It has also been proposed that seasonal fluctuations in host immunity may periodically change host susceptibility to influenza infections [7]. However, to our knowledge, all models assessing disease burdens of influenza assumed constant effects of influenza over the study period [1,2,8] and no studies have investigated the seasonal effects of influenza on mortality.

Hong Kong has a typical subtropical climate with well separated four seasons, and the average temperature is about 24°C. In this study we applied a time-varying coefficient Poisson regression model to the weekly counts of deaths, using the laboratory virology surveillance data in Hong Kong to explore the seasonal effects of influenza on mortality. Our findings suggest that influenza has significant seasonal effects in terms of influenza-associated mortality from respiratory and cardiovascular diseases.

## Methods

### Data

We obtained weekly numbers of mortality during the period of 1996 to 2002 from the Hong Kong Census and Statistics Department, for five underlying causes of death: all-cause (excluding accidental deaths), cardiovascular and respiratory diseases (CRD) (International Classification of Diseases, Ninth Revision [ICD-9] codes 390–519 and Tenth Revision [ICD-10] codes I00-I99, J00-J98), and sub-categories pneumonia and influenza (P&I) (ICD-9 codes 480–487; ICD-10 codes J10-J18), chronic obstructive pulmonary disease (COPD) (ICD-9 codes 490–496; ICD-10 codes J40-J47), cerebrovascular diseases (ICD-9 430–438; ICD-10 codes I60-I69) and ischemic heart disease (IHD) (ICD-9 codes 410–414; ICD-10 codes I20-I25). We did not adjust for a potential shift in disease counts due to the switch of coding system from the ICD-9 to ICD-10 since this shift was estimated to be small [9].

We obtained the virology surveillance data from the microbiology laboratory of Queen Mary Hospital (QMH). This laboratory routinely collects specimens from outpatients and inpatients in the hospitals and clinics around the Hong Kong Island, to test for six respiratory viruses including influenza virus (types A and B), respiratory syncytial virus (RSV), adenovirus and parainfluenza (PIV, types PIV-1, PIV-2 and PIV-3) [10]. We used the weekly proportions of specimens tested positive for each virus, to represent their activities in Hong Kong population. The QMH data are part of the sentinel surveillance implemented by the Government Virus Unit in the Department of Health (DH) which covers the entire territory of Hong Kong, but the DH data are only available after 1998 and specimens are tested only for influenza viruses. In fact the specimens from QMH account for around 40% of total specimens in DH surveillance and the weekly proportions of specimens positive for influenza from QMH and DH are highly correlated (Spearman coefficient 0.8, for data during 1998–2002). Given the homogeneity of Hong Kong population, we therefore believe that the data from the QMH could represent the virus activity in the entire Hong Kong. Meteorological data were obtained from the Hong Kong Observatory [11].

### Statistical modeling

For each disease category, we applied a semi-parametric Poisson regression method under the framework of generalized additive model (GAM) [12], to the weekly numbers of mortality. We first built the core model:(1)

Here *μ*_*t *_is denoted as the expected number of mortality at week *t*. The seasonality of mortality, temperature and humidity were adjusted for as confounders in the core model, defined by natural spline smoothing functions (*NS*) of consecutive week numbers *t*, weekly arithmetic means of temperature (*temp*) and relative humidity (*humd*) at week *t*, respectively. The choice of degrees of freedom (*df*) for each smoothing function was based on the adequacy of the core model judged by the criteria that the partial autocorrelation function plots of the residuals are with +/- 0.1 and do not exhibit any discernible patterns as described in a previous study [10]. Over-dispersion was adjusted for by a quasi-likelihood estimation method [13].

The weekly proportions of specimens positive for influenza viruses were then entered into the core model, along with the proportions of RSV, adenovirus, PIV-1, PIV-2 and PIV-3 at the current week, to calculate the effects of influenza with adjustment for co-circulation of other respiratory viruses. A typical form of this main effect model is(2)

where *flu*_*t*-*l *_is the proportion of specimen positive for influenza A and B at the lag *l *week (*l *= 0, 1, 2, 3) of week *t*. For the subsequent analyses we used the best lag week, which had the smallest *p*-value for coefficients *β *among the four models of different lag weeks for each outcome.

To evaluate the seasonal effects of influenza, we applied two different methods. The first method is time-varying coefficient Poisson model [14]. As influenza activity tends to reach two peaks a year in Hong Kong [10], we decided to add two pairs of sinusoidal terms to capture these two peaks, as shown below:(3)

Here *k *= 1 is to model the annual pattern of seasonal effects and *k *= 2 to capture the semiannual pattern. In order to assess whether there is a seasonal effect of influenza, we performed a likelihood ratio test by calculating the difference of statistical deviance between the time-varying coefficient Poisson model (equation 3) and the main effect model (equation 2) [12]. To further justify our selection of Poisson model with two pairs of sinusoidal terms, we also used likelihood ratio tests to compare two-cycle model (two pairs of sinusoidal terms, *k *= 1, 2 in equation 3) with one-cycle model (one pair, *k *= 1 in equation 3).

The second method is to estimate effects of influenza in four seasons respectively, using the conventional interaction model. We added into the core model (equation 1) the interaction terms between influenza and seasons, which were defined by products of *flu*_*t*-*l *_with each of three season dummy variables. The season dummy variables were defined as follows: week 9–21 as spring, week 22–34 as summer, week 35–47 as autumn and week 48-week 8 as winter. This definition follows the Hong Kong Observatory's definitions of four seasons as March–May, June–August, September–November and December–February [15]. The general form of interaction model is(4)

*I*_winter _was set to 1 for winter and 0 for otherwise, and similar definitions were applied for *I*_spring _and *I*_summer_.

We then calculated weekly influenza-associated excess risks (ER) per inter-quartile range (IQR) increase of influenza isolation proportions (12.3% in our data) as a measurement for the seasonal effects of influenza on mortality, by taking anti-logarithm transformation of sum of coefficients for the *flu *term in the time-varying coefficient Poisson model (equation 3, with *t *= 1, 2,...52) after multiplying them with 12.3%, and subsequently deducting by one.

Similarly, the excess risk for each season was derived from *γ *coefficients of interaction model (equation 4). Weekly influenza-associated excess risks represent the increase of risk for influenza associated deaths when the influenza proportions increase from zero to 12.3%. To compare with previous studies, we also calculated the influenza associated excess mortality, which was defined as the difference between observed numbers of deaths and baseline deaths in the absence of virus activity [1].

We performed three sensitivity analyses. We adjusted for potential confounding of temperature by adding the lag 1 difference of weekly geometric mean, where lag 1 difference was defined as current week minus previous week, into the core model (equation 1). We also tested the lag effects on the seasonal effects of influenza, by replacing the proportions of influenza isolates at the best lag week by those at four different lag weeks (up to three weeks). To exclude the possibility that seasonal effects of influenza were caused by the inadequate adjustment for co-circulation of RSV, we performed the third sensitivity analysis with additional variables for interaction between two pairs of harmonic terms and proportions of RSV positive isolates. We used the statistical package R for all the analyses [16].

## Results

Weekly average temperature, relative humidity and proportions of specimens positive for respiratory viruses are summarized in Table 1. On average, proportions of positive isolates for influenza viruses were lowest in autumn, but at a similar level in other seasons. More RSV viruses were isolated in spring and summer, and more PIV-1 in autumn and winter. Adenovirus, PIV-2 and PIV-3 did not exhibit any discernible seasonal difference. More deaths were recorded in winter for all the disease categories.

**Table 1:** Season averages of weekly meteorological data, proportions of positive specimens and numbers of death

	Whole year		Autumn		Winter		Spring		Summer	
	
Variables	Mean	(SD)	Mean	(SD)	Mean	(SD)	Mean	(SD)	Mean	(SD)
Temperature	23.7	(4.7)	25.6	(2.7)	17.6	(2.4)	22.9	(3.0)	28.6	(1.1)
Humidity	78.0	(7.8)	74.5	(8.3)	74.1	(8.9)	82.0	(5.2)	81.3	(3.9)

Virus isolation (%)										
Influenza	10.1	(10.1)	2.0	(2.7)	13.2	(14.0)	13.0	(9.0)	12.0	(6.2)
RSV	8.8	(6.8)	8.7	(7.1)	3.0	(3.3)	10.6	(5.7)	12.8	(6.4)
Adenovirus	4.2	(3.4)	3.7	(2.9)	4.1	(2.7)	4.2	(3.9)	4.9	(3.8)
PIV-1	1.5	(2.6)	2.4	(3.4)	2.3	(3.2)	0.5	(0.8)	0.6	(1.2)
PIV-2	0.3	(0.6)	0.5	(0.7)	0.3	(0.5)	0.1	(0.4)	0.3	(0.5)
PIV-3	2.8	(2.1)	2.9	(2.5)	2.4	(1.9)	3.2	(2.0)	2.6	(2.0)

Mortality										
All-cause										
All ages	623.3	(65.1)	572.7	(38.6)	682.6	(59.4)	644.7	(58.2)	593.4	(34.2)
65+	468.5	(61.3)	420.0	(38.3)	524.6	(56.7)	488.0	(53.2)	441.3	(31.0)
CRD										
All ages	278.4	(48.8)	238.8	(21.9)	321.5	(46.3)	296.5	(46.7)	256.8	(23.5)
65+	242.7	(45.0)	205.8	(20.9)	284.9	(41.4)	258.4	(41.9)	221.7	(19.9)
P&I										
All ages	65.0	(15.8)	56.8	(13.4)	70.4	(15.4)	67.0	(15.6)	65.9	(15.4)
65+	59.5	(14.3)	52.2	(12.4)	64.8	(13.5)	61.1	(14.3)	59.9	(14.1)
COPD										
All ages	40.7	(12.2)	30.4	(5.9)	47.2	(13.1)	47.3	(11.3)	38.1	(7.8)
65+	37.1	(11.3)	27.4	(5.6)	43.8	(11.9)	43.0	(10.6)	34.3	(6.9)
Cerebrovascular										
All ages	61.9	(13.5)	55.0	(9.2)	72.2	(12.6)	66.0	(13.5)	54.5	(8.9)
65+	52.8	(12.6)	46.0	(8.6)	61.7	(11.7)	57.2	(12.6)	46.0	(8.6)
IHD										
All ages	62.4	(13.9)	54.2	(8.5)	74.5	(14.3)	65.5	(13.0)	55.6	(7.6)
65+	53.6	(12.8)	46.0	(7.6)	65.7	(12.7)	55.8	(12.0)	47.0	(6.9)

The likelihood ratio tests, which compared the two-cycle with the one-cycle Poisson models, showed that the two-cycle models better captured the seasonal effects of influenza, with the only exception of mortality with underlying cause of pneumonia and influenza. However the one-cycle model also did not fit this disease category, suggesting that the effects of influenza on pneumonia and influenza mortality are unlikely to have any seasonal patterns (data not shown).

**Figure 1:**
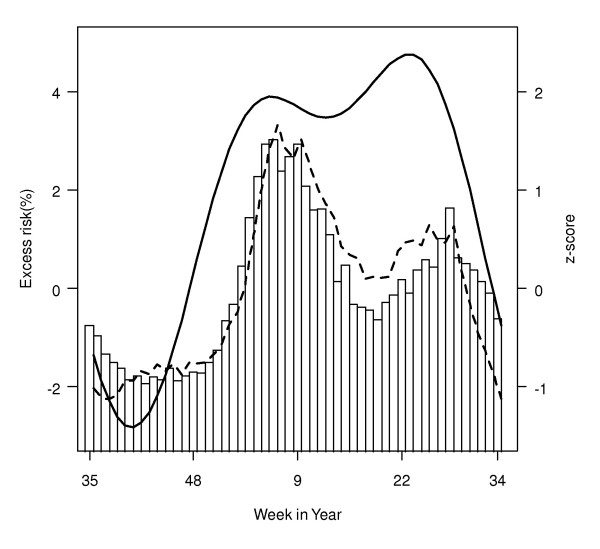
**Seasonal patterns of excess risks, excess mortality associated with influenza and proportions of influenza isolates**. Excess risks (thick line) associated with per IQR increase of influenza virus activity for all-cause mortality in the all-ages group were calculated after controlling for influenza virus seasonality. Weekly excess all-cause mortality is plotted in broken line and proportions of influenza are shown in bar. Weekly excess mortality and proportions of influenza isolates were Z-score standardized by each year and then averaged over seven years.

The seasonal effects of influenza were measured by variations in weekly influenza-associated excess risks per IQR increase of influenza virus activity. For all-cause mortality in the all-ages group, the seasonal effects showed an evident two-peak pattern, with the first in winter (around week 1) and the second in late spring or early summer (around week 22) (Figure 1). The excess mortality, in a standardized z-scores which were averaged over the seven years of study period, was higher in winter and had a pattern similar to virus activity but different from seasonal effects of influenza (Figure 1).

**Figure 2:**
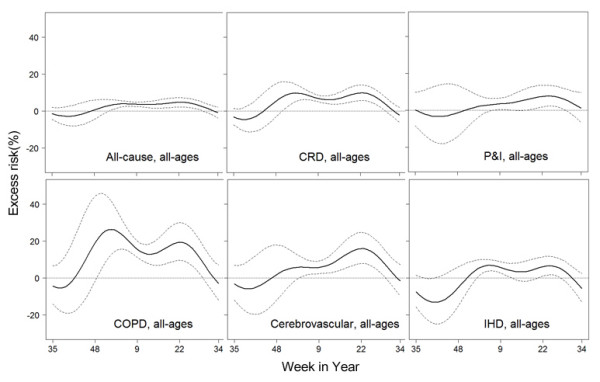
**Excess risks of mortality at the best lag week for the all-ages group**. Excess risks associated with per IQR increase of influenza virus activity are plotted in solid line. Broken lines represent 95% confidence intervals.

The two-peak pattern of seasonal effects of influenza was found for all the disease categories in the all-ages group (Figure 2). Particularly, for COPD, the seasonal effects of influenza were characterized with a sharp winter peak; whereas for cerebrovascular diseases the spring/summer peak was more evident. Similar patterns for the seasonal effects of influenza were observed in the elderly group, with slight changes in magnitudes (see Additional file 1). The seasonal effects of influenza on mortality were statistically significant (*p *< 0.05) for cardiovascular and respiratory diseases, COPD, cerebrovascular diseases, ischemic heart diseases as well as all-cause, in both 65+ and all-ages groups. But there was little evidence that the seasonal effects of influenza on mortality for pneumonia and influenza also had a two-peak pattern, although the total effects of influenza were highly significant (Table 2).

**Table 2:** Excess risks of mortality per IQR increase of influenza virus activity during four seasons

Disease	lag	Total	Autumn	Winter	Spring	Summer	*p *value ‡
All-cause							
all ages	1	3.5 (2.4,4.6)	-0.9 (-5.6,4.2)	3.9 (2.6,5.3)	3.9 (2.5,5.3)	2.3 (0.3,4.3)	<0.001
65+	1	4.3 (3.0,5.6)	-1.4 (-7.0,4.5)	4.5 (3.0,6.1)	4.5 (2.9,6.1)	3.8 (1.4,6.3)	0.005
CRD							
all ages	2	6.5 (4.8,8.2)	-1.2 (-7.4,5.4)	8.0 (5.8,10.2)	6.5 (4.5,8.6)	3.6 (0.4,7.0)	<0.001
65+	1	6.7 (4.9,8.5)	-2.0 (-10.0,6.8)	7.3 (5.2,9.6)	7.0 (4.7,9.4)	5.0 (1.5,8.5)	<0.001
P&I							
all ages	1	4.2 (1.5,6.9)	1.0 (-11.9,15.8)	4.2 (0.9,7.7)	3.0 (-0.5,6.5)	5.8 (0.8,11.0)	0.224
65+	1	4.3 (1.6,7.1)	0.6 (-12.6,15.8)	4.1 (0.7,7.6)	2.8 (-0.7,6.4)	6.7 (1.6,12.1)	0.156
COPD							
all ages	2	14.9 (11.0,19.0)	-7.0 (-20.3,8.5)	18.8 (13.8,24.1)	14.3 (9.6,19.2)	9.4 (1.8,17.6)	<0.001
65+	2	13.7 (9.7,17.9)	-9.0 (-22.6,6.9)	17.0 (11.9,22.3)	13.1 (8.3,18.1)	9.7 (1.7,18.3)	<0.001
Cerebrovascular							
all ages	1	6.3 (3.2,9.5)	-1.9 (-14.9,13.2)	5.5 (1.9,9.3)	8.3 (4.3,12.4)	6.1 (0.0,12.5)	0.001
65+	1	7.3 (3.9,10.9)	-3.8 (-17.9,12.7)	6.2 (2.2,10.4)	10.4 (5.9,15.0)	6.6 (-0.1,13.8)	0.001
IHD							
all ages	0	6.0 (3.5,8.6)	0.9 (-12.3,16.2)	6.3 (3.4,9.3)	6.7 (3.3,10.2)	4.4 (-0.3,9.3)	0.021
65+	0	5.6 (2.9,8.3)	-2.9 (-16.7,13.2)	5.8 (2.7,9.1)	5.9 (2.3,9.7)	4.4 (-0.7,9.8)	0.015

The 95% confidence intervals of excess risks are shown in brackets.

‡ *p *value of likelihood ratio tests, indicating significance of seasonal variation

Consistent with the results from the seasonal effect models, the influenza effects in four seasons estimated from the interaction models (measured by influenza-associated excess risks in each season) were found lowest and not significant in autumn for all the age-disease categories (Table 2). The influenza effects on mortality in four seasons were estimated to be higher in both winter and spring for cardiovascular and respiratory diseases, ischemic heart diseases and all-cause, whereas the influenza effects was the highest in winter for COPD, in spring for cerebrovascular diseases and in summer for pneumonia and influenza, respectively.

In overall, the ER for all-cause mortality associated with per IQR increase in proportions of influenza positive specimens was 3.5% (95% CI, 2.4–4.6%) in the all-ages group and 4.3% (95% CI, 3.0–5.6%) in the 65+ age group. When proportions of positive specimens increased 12.3%, the risks of death were 6.5%, 4.2%, 14.9%, 6.3% and 6.0% higher for cardiorespiratory diseases, pneumonia and influenza, COPD, cerebrovascular diseases and ischemic heart diseases, respectively.

The sensitivity analysis replacing the arithmetic mean of temperature by the lag 1 difference of its weekly geometric mean (i.e. mean of current week minus that of last week) did not significantly change the patterns of seasonal effects of influenza for all disease categories (See Additional file 2). The two-peak patterns of seasonal effects of influenza were robust to different lag weeks, but slightly varying in magnitude and timing of peaks (See Additional file 3). Adjustment for seasonal effects of RSV in mortality models slightly changed the magnitudes of influenza effects, but in overall did not alter the two-peak patterns (See Additional file 4).

## Discussion

This study for the first time demonstrated that the effects of influenza on mortality have a seasonal pattern. We adopted influenza associated excess risk as a measurement for the seasonal effects of influenza, which allows us to evaluate the changes in mortality risks attributable to influenza around the year independent of the seasonal variations of virus activity. Seasonal effects of influenza in this study are not equivalent to influenza associated excess mortality, which has been widely used to measure the disease burden of influenza [17]. Influenza associated excess mortality is determined not only by the effects of influenza, but also by the levels of virus activity. The excess risk is also different from the virus activity: the former measure the severity of diseases given influenza infections, but the latter probability of infections. The virus activity is controlled by transmission efficiency of viruses as well as by the herd immunity, but the probability to develop into a severe disease is more likely affected by the pathogenicity of viruses and individual immunity levels against respiratory pathogens including influenza virus and pneumococcus bacteria, the latter of which often cause secondary pneumonia. This study also provides a valid and simple modeling strategy with proper adjustment for confounding of environmental factors and co-circulation of other respiratory viruses, which can be applied to other regions.

We found that the effects of influenza on mortality exhibit a similar two-peak pattern as the virus activity. The spread of influenza increases the mortality risks of cardio-respiratory diseases to a greater extent in winter and late spring/summer than the rest of year. This pattern is probably the result of interaction between host susceptibility and environmental factors such as temperature. Both cold and hot weather have been linked to increased cardio-respiratory mortality in numerous studies [18-20]. It has been proposed that cold air could cool the upper respiratory tract epithelium and thereby slow down mucociliary clearance [21]. Exposure to low and high temperature can both increase blood viscosity and trigger cardiovascular events [22]. Influenza infections have similar prothromobotic and proinflammatory effects [23]. Numerous studies in the temperate regions showed that exacerbations of COPD frequently occur in cold temperature when influenza viruses tend to be active, as a result of stimulated airway inflammation [24,25]. Similar to the seasonal effects of influenza, the mortality counts of all-cause deaths also exhibited a two-peak pattern. Usually one big winter spike usually appearing weeks after when the lowest temperature was recorded and a small spike also appeared soon after the week when the highest temperature recorded in summer (data not shown). The least death numbers recorded in autumn. This mortality seasonality was also reported in other subtropical cities [22]. Furthermore, a multicity study in the United States demonstrated that the temperate effects on mortality exhibited a U-shape pattern with a turning point around 26°C for the southern cities [26]. Similarly, influenza viruses tend to be active in winter and early summer. Overlapping in timing of peaks between influenza activity and the temperature effects on mortality, plus the similar biological effects of viruses and extreme temperate, suggest that the synergistic interaction between influenza and temperature is highly plausible.

Host immunity could also follow a seasonal variation, although studies for seasonal changes of human resistance to influenza remain controversial [7]. Some reports suggest that the immune defenses are elevated during summer and weakened during winter, whereas others drew the opposite conclusion. This discrepancy likely originated from different sampled subjects (patients or healthy subjects) and unmeasured confounding factors such as perception of stress [27]. It has been suggested that photo-period and levels of melatonin and vitamin D play an important role in regulating the seasonal rhythm of human immunity, but again evidence is rather limited [9]. More research is needed in order to fully understand the seasonal fluctuation of influenza effects on health.

According to our results, the peaks of seasonal effects of influenza on mortality appear to precede those of virus activity by weeks (Figure 1). Interestingly, we found the consultation rates of influenza-like illness in private doctors also preceded virus activity by an average of four weeks [28]. This coincidence may suggest that the virulence of influenza viruses probably reaches its peak at the beginning of an outbreak and may diminish later, as the population gradually gains the herd immunity against novel strains. The virulence of influenza is determined by both the host and virus [29]. In the beginning of their emergence, the new virus strains introduced by frequent antigenic drifts could replicate more efficiently in the immunologically naïve host and as a result impose a more serious threat on the community health [30]. We proposed that the seasonal changes of influenza virulence might reflect the frequent antigenic shifts of viruses within an influenza epidemic season. Several large-scale phylogenetic studies could not find evidence of local evolution of virus strains in the temperate regions and proposed that the subtropics and tropics, especially East and Southeast Asia, are more likely the virus reservoirs where the reassortment of co-circulating lineages could occur to result in emerging novel virus strains that later spread to the temperate regions [31-33]. The better understanding to the mechanisms of influenza virus evolution must base on the more comprehensive surveillance networks in the tropics and subtropics.

As H3N2 is believed to cause more mortality and to spread more efficiently than H1N1 and B [34,35], we hypothesize that seasonal effects of influenza could be a result of different subtypes dominating over different seasons. Because the virus subtype data during the study period are not available from QMH, we obtained from the Government Virus Unit of the Department of Health in Hong Kong the weekly proportions of specimens positive for influenza H3N2, H1N1 and B from 1998 to 2002. Overall, the proportions of H3N2 isolates in all influenza virus positive isolates show a similar two-peak pattern to the seasonal variation of influenza effects from winter to summer, with a higher peak in summer than in winter (See Additional file 5). We then applied the time-varying coefficient Poisson model to the weekly death numbers from 1998 to 2002 when the subtype data were available in Hong Kong, with the variables for weekly proportions of subtype H3N2 and for the product of two sinusoidal pairs and H3N2 proportions added into the model to measure the seasonal effects of H3N2. The proportions of H1N1 and type B were also entered into the model. The results suggested that H3N2 contributed to the majority of seasonal effects of influenza, as the excess risks associated with H3N2 showed similar patterns as those associated with the combined proportions of three types/subtypes of influenza viruses (data not shown). We repeated the above analyses separately for H1N1 and type B viruses and found that none of them has significant seasonal variations. However, the lack of seasonal effects in H1N1 and B could be the result of mild activities of these two types/subtypes during our study period. Nevertheless, we could not rule out that changing dominance of different virus subtypes could also contribute to seasonal effects of influenza.

The seasonal effects are significant for chronic conditions but not for the mortality of pneumonia and influenza which is considered as the most specific endpoint of influenza infection [1,10]. We think this could be due to the fact that we used the underlying cause of death, which probably results in underreporting of pneumonia and influenza cases and subsequently underestimating of influenza associated pneumonia and influenza mortality. The effects of influenza on chronic conditions, especially COPD, decrease promptly after reaching the peaks, thereby exhibiting more pronounced seasonal variations than the seasonal effects on pneumonia and influenza. We speculate that there is probably a "harvesting" effect for influenza associated mortality [36]. The vulnerable people, who had been suffering from chronic diseases, would die soon after infection and their causes of deaths would likely be recorded as their preexisting conditions. At the beginning of an epidemic, influenza infections probably claim the most vulnerable people to empty the susceptibility pool, leading to a subsequent decline in the influenza effects. Such a temporal change in the susceptibility pool may not be evident for those who had been previously healthy and were grouped as pneumonia and influenza deaths.

There are several caveats in our study. Similar to our previous studies for health burden of influenza, we adopted the best lag week, which offered the most significant association between influenza activity and mortality outcome. Sensitivity analyses of different lag weeks showed that although magnitudes of peaks are somehow sensitive to the lag weeks, the two-peak pattern of seasonal effects of influenza is relatively robust to the changes of lag weeks. We did not take account of the potential shift in mortality counts between year 2000 and 2001 caused by switch of ICD-9 to ICD-10, however this shift may be small and only affect the temporal variations of mortality data during a short period. In addition, we must be cautious to apply our findings to other tropical and subtropical regions, because unlike most of other tropical and subtropical regions, Hong Kong is a highly developed and compact urban area. Nevertheless, future studies in the temperate and developing tropical and subtropical regions may help us better understand the mechanisms behind the seasonal effects of influenza.

## Conclusion

In summary, this study demonstrated that the effects of influenza on mortality show a seasonal pattern. The seasonal variation in influenza effects on mortality could provide strong evidence for the need of refining influenza control policy and promoting public awareness to outbreaks of influenza both in the warm and cold seasons.

## Abbreviations

SD: standard deviation; RSV: respiratory syncytial virus; PIV: parainfluenza virus; CRD: cardiovascular and respiratory diseases; P&I: pneumonia and influenza; COPD: chronic obstructive pulmonary disease; IHD: ischemic heart diseases; IQR: inter-quartile range.

## Competing interests

The authors declare that they have no competing interests.

## Authors' contributions

The authors LY and CMW designed the study and analyzed the data. KPC, YKC, CQO and JSMP provided the interpretation and public health implication for the results. KHC and JSMP provided and interpreted the virology data. LY and CMW wrote the first draft. LY, CMW and JSMP finalized the paper. All authors read and approved the final manuscript.

## Pre-publication history

The pre-publication history for this paper can be accessed here:

http://www.biomedcentral.com/1471-2334/9/133/prepub

## Supplementary Material

Additional file 1 **Excess risks of mortality at the best lag week for the 65+ group**. Excess risks associated with per IQR increase of influenza virus activity are plotted in solid line. Broken lines represent 95% confidence intervals. Click here for file

Additional file 2 **Excess risks for mortality of the all-ages group with different adjustments for temperature**. Excess risks associated with per IQR increase of influenza virus activity at the best lag week after adjustment for weekly arithmetic mean of temperature are shown in solid line and those after adjustment for weekly geometric mean of temperature in broken line. Click here for file

Additional file 3 **Excess risks for mortality at different lag weeks**. Solid line shows excess risks associated with per IQR increase of influenza virus activity at current week (lag 0), dotted line shows lag 1 week, long dash line lag 2 weeks and two dash line lag 3 weeks. The thick line represents the estimates for the best lag week. Click here for file

Additional file 4 **Excess risks for mortality without and with adjustment for seasonal variation of RSV effects**. Solid line represents excess risks associated with per IQR increase of influenza virus activity without adjustment for seasonal variation of RSV effects and broken line represents excess risks with adjustment for seasonal variation of RSV effects. Click here for file

Additional file 5 **Average weekly proportions of H3N2, H1N1 and B in all influenza isolates, 1998 – 2002**. The data were obtained from Department of Health. Click here for file
