# Supplementary figures and images for: Seasonal effects of influenza on mortality in a subtropical city
Source: BMC Infect Dis. 2009 Aug 22;9:133. doi: 10.1186/1471-2334-9-133 (PMC2739210; doi:10.1186/1471-2334-9-133)

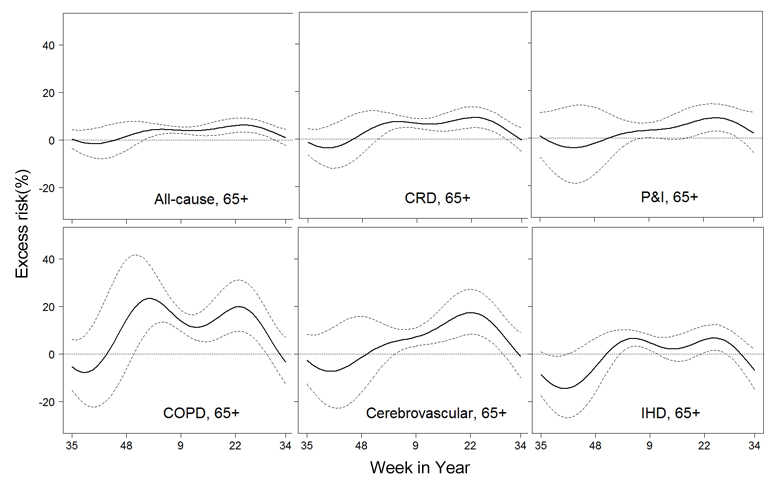

Supplement: Additional file 1 — Excess risks of mortality at the best lag week for the 65+ group. Excess risks associated with per IQR increase of influenza virus activity are plotted in solid line. Broken lines represent 95% confidence intervals. [file 1471-2334-9-133-S1.jpeg]

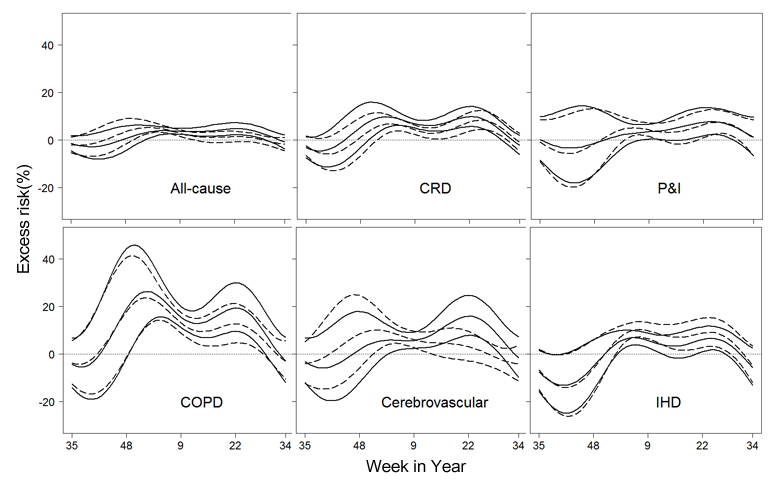

Supplement: Additional file 2 — Excess risks for mortality of the all-ages group with different adjustments for temperature. Excess risks associated with per IQR increase of influenza virus activity at the best lag week after adjustment for weekly arithmetic mean of temperature are shown in solid line and those after adjustment for weekly geometric mean of temperature in broken line. [file 1471-2334-9-133-S2.jpeg]

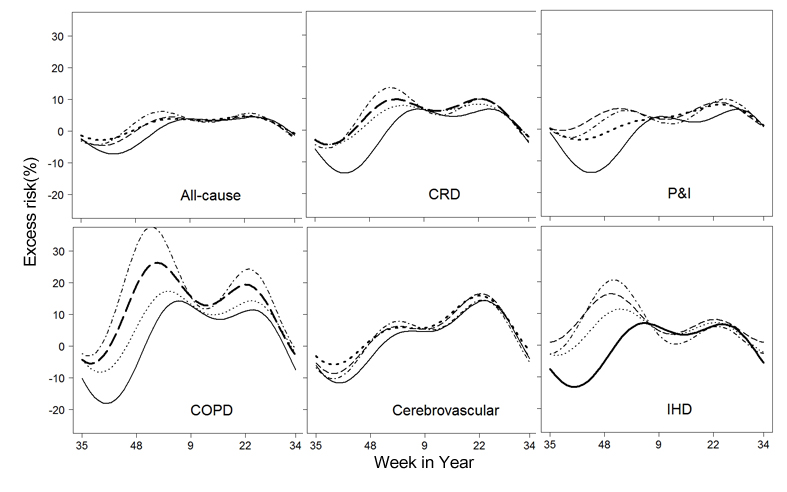

Supplement: Additional file 3 — Excess risks for mortality at different lag weeks. Solid line shows excess risks associated with per IQR increase of influenza virus activity at current week (lag 0), dotted line shows lag 1 week, long dash line lag 2 weeks and two dash line lag 3 weeks. The thick line represents the estimates for the best lag week. [file 1471-2334-9-133-S3.jpeg]

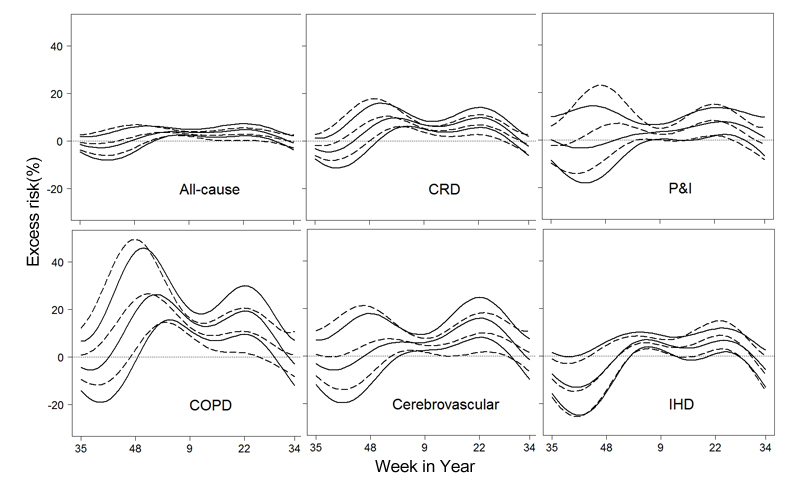

Supplement: Additional file 4 — Excess risks for mortality without and with adjustment for seasonal variation of RSV effects. Solid line represents excess risks associated with per IQR increase of influenza virus activity without adjustment for seasonal variation of RSV effects and broken line represents excess risks with adjustment for seasonal variation of RSV effects. [file 1471-2334-9-133-S4.jpeg]

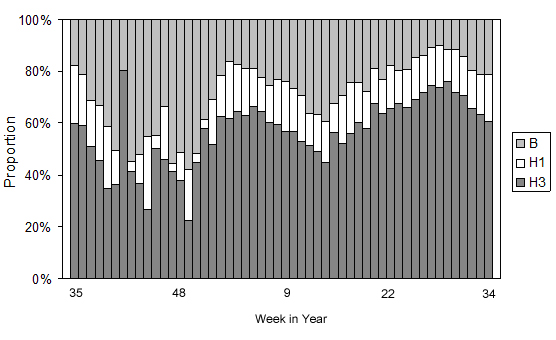

Supplement: Additional file 5 — Average weekly proportions of H3N2, H1N1 and B in all influenza isolates, 1998 – 2002. The data were obtained from Department of Health. [file 1471-2334-9-133-S5.jpeg]
